# Calculation of horizontal displacement of loess fill slope supported by frame prestressed anchors based on minimum potential energy method

**DOI:** 10.1038/s41598-022-15473-3

**Published:** 2022-07-04

**Authors:** Zhuangfu Zhao, Yanpeng Zhu, Shuaihua Ye

**Affiliations:** 1 School of Civil Engineering, Lanzhou University of Technology, Lanzhou, 730050 China; 2 Key Laboratory of Disaster Mitigation in Civil Engineering of Gansu Province, Lanzhou University of Technology, Lanzhou, China

**Keywords:** Civil engineering, Natural hazards

## Abstract

Combined with the deformation characteristics of flexible retaining structure, the horizontal displacement calculation method of loess fill slope supported by frame prestressed anchors is proposed. Based on the minimum potential energy method, the analytical solution of horizontal displacement of slope under self-weight and additional load is derived, and the influence of soil parameters and supporting structure parameters on displacement is analyzed. The proposed calculation method is applied to a practical engineering and compared with the numerical simulation, which shows that the method is reasonable and reliable. The minimum potential energy method is clear in concept and simple in solving the horizontal displacement of loess fill slope supported by frame prestressed anchors. The calculation method proposed in this paper can be applied to the structural optimization design of loess fill slope supported by frame prestressed anchors, and further enrich the displacement calculation theory of slope supported by flexible retaining structure.

## Introduction

With the acceleration of urbanization construction, the contradiction between the large-scale demand for construction land and the scarcity of land resources becomes increasingly prominent, which leads to the emergence of large-scale land reclamation projects by cutting mountains and filling gullies. More than 700 hills were leveled in Lanzhou, Gansu Province, China, and the leveling soil was about 25 km^2^. Shiyan, Hubei Province, China has carried out a 150,000-mu mountain cutting and land reclamation project. Yan' an New District, Shaanxi Province, China has started the largest loess filling project in the world. It is planned to cut mountains and fill gullies in about 90 km^2^ within ten years^1,2^. At the same time, the number of airport construction projects in hilly and gully areas is increasing, and the record of high fill height is constantly refreshed. In the large-scale land reclamation project of cutting mountains and filling gullies and the airport construction project on the loess ridge terrain, a large number of construction modes using fill to make up the engineering land have appeared^3,4^. Most of the original foundations of fill slopes are slope foundations, some of them are deformed and damaged^5,6^. The loess area is often characterized by broken terrain and ravines. With the construction gradually expanding to higher places and mountainous areas, due to the limitation of terrain conditions, it can be predicted that high fill slopes will appear in large quantities in future construction projects in loess areas, and it is urgent to study its deformation characteristics and failure mechanism.

In view of the high fill slopes with different fillers and different filling technologies, some scholars have done a lot of work in design methods, filling methods and slope stability research, and obtained useful research results^7,8^. For the fill slope in loess area, the filled loess is both the settlement medium and the load of the underlying stratum, which is prone to consolidation settlement and collapse settlement under the action of self-weight load and additional load on the upper part^9–11^. Because the design theory of high fill slope is not mature, it is easy for high fill slope to have large horizontal and vertical displacement at the top of the slope and shear failure at the foot of the slope in the later operation process. In severe cases, the slope will slide directly or even collapse, which will seriously affect the safety of people's lives and property^7,8,12^. In order to avoid the occurrence of engineering accidents, in addition to controlling the filling quality, it is also necessary to carry out the retaining reinforcement for the fill slope. The frame prestressed anchor is a flexible retaining structure with active support. Because of its light weight and good reinforcement effect, it is widely used in the slope support and reinforcement design. By applying certain prestress, the advance reinforcement of the slope can be realized, and the sliding and deformation of the slope can be effectively constrained and controlled^13,14^. Although there are many research results in the displacement control and settlement prediction method of filling^2,4,6^, the displacement deformation characteristics and failure mechanism of the loess fill slope are not fully understood, and the displacement calculation method of loess fill slope supported by frame prestressed anchors remains to be further studied.

In this paper, the loess fill slope supported by frame prestressed anchors is taken as the research object. Considering the deformation characteristics of flexible retaining structure, the displacement calculation model of slope is established by analyzing the forces acting on the supporting structure and considering the influence of various supporting components on slope deformation. The analytical solution of horizontal displacement of slope is derived based on the minimum potential energy method, and the influence of soil parameters and supporting structure parameters on slope displacement is analyzed. Finally, the method is verified by practical engineering application and numerical simulation. The method proposed in this paper can optimize the structural design of loess fill slope supported by frame prestressed anchors, and further enrich the displacement calculation theory of the slope supported by the flexible retaining structure.

## Establishment of horizontal displacement calculation model.

### Mechanical analysis of supporting structure

At present, the frame prestressed anchor structure has been widely used in slope engineering and foundation pit engineering. In filling engineering, the flexible retaining structure of frame prestressed anchor has also played a considerable role. In the foundation pit engineering, considering the space effect, the displacement and deformation of the supporting structure of frame prestressed anchor is assumed to be the constrained torsion problem, according to the elastic stability theory, some scholars have given the deformation curve equation of the retaining wall of frame prestressed anchors^15,16^:

1 $$ y = \frac{s}{2}\sin \left( {\frac{\pi x}{L}} \right)\left( {1 - \cos \frac{\pi z}{H}} \right) $$

where, *y* is the horizontal displacement of retaining wall at *z* from the foot of foundation pit. *x* is the distance from the foundation pit corner. *z* is the calculated height. *L* is the calculated length of foundation pit, $s$ is the maximum horizontal displacement in the middle of the calculated length. *H* is the height of foundation pit.

However, in slope engineering, the problem of space effect is seldom considered. It is considered that the deformation of slope can be considered as a plane strain problem. In order to simplify the calculation, it is assumed that the deformation of frame beams and columns conforms to the assumption of plane section, and the deformation of frame (beams and columns) is mainly bending deformation. According to the related research^17^, it can be assumed that the frame beam and the frame column can be decomposed into independent continuous beam units without considering the torsional deformation of the beam. Because the frame column is the main stress component, the frame beam only plays the role of spatial coordination, so it is only necessary to consider the bending deformation of the frame column and ignore the bending deformation of the frame beam.

**Figure 1:**
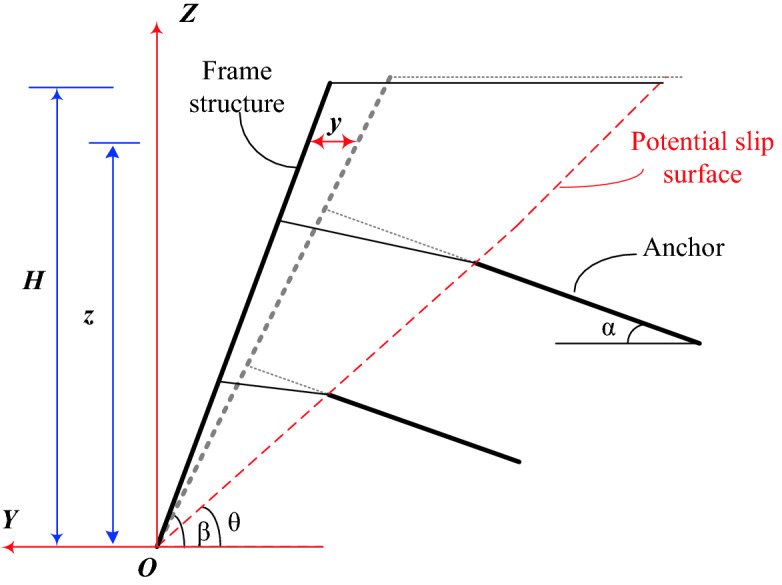
Horizontal displacement calculation model.

Based on this, the calculation model of horizontal displacement of loess fill slope supported by frame prestressed anchor flexible supporting structure, as shown in Fig. 1, is established. According to the research results of Zhou and Zhu^18^, the short pile foundation with a length of 3–5 m is generally set at the bottom of the supporting structure of the frame prestressed anchors, and its function is mainly to increase the horizontal thrust resistance. Considering the passive earth pressure of the soil below the slope bottom, it is considered that the horizontal displacement of the supporting structure at the slope bottom is approximately 0. Assuming that the maximum displacement of the slope occurs near the top of the slope. For convenience of calculation, the maximum displacement of the retaining wall at the top of the slope is *s*. On the basis of Eq. (1), the deformation curve equation of retaining wall is simplified as follows:

2 $$ y = \frac{s}{2}\left( {1 - \cos \frac{\pi z}{H}} \right) $$

where, *y* is the horizontal displacement of retaining wall at *z* from the foot of fill slope. *z* is the calculated height. *H* is the height of fill slope. That is, in slope engineering, the structural deformation is only analyzed in plane without considering the effect of the calculated length *L*.

The filling body of loess fill slope produces horizontal lateral displacement under the action of upper additional load and self-weight of soil, and produces a slight fold step deformation at the slip surface (Fig. 1). The prestressed anchors have tensile and shear deformation, and the frame column has bending deformation. On the whole, the forces borne by the supporting structure mainly composed of the active earth pressure behind the slope, the self-gravity, and the prestress of anchors, among which the self-weight of retaining structure has little influence on slope deformation and can be ignored. Therefore, the total potential energy of fill slope system supported by frame prestressed anchors includes the bending strain energy of frame columns, the tensile strain energy of prestressed anchors, the shear strain energy of prestressed anchors, the tensile external potential energy of anchors and the external potential energy of active earth pressure.

### Analysis of the tension force of anchors

As a load-transfer member, the anchor transmits the self-supporting pull-out force of the soil, and provides the pull-out force through the soil anchor effect. According to Zhou’s research^19^, assuming that there is no prestress loss, the tensile force of the anchor is:

3 $$ T_{j} = (T_{j1} + T_{j2} )\cos \alpha $$

where $T_{j1}$ is the pullout resistance provided by the *j-th* anchor (the pullout resistance provided by the soil anchor effect). $T_{j2}$ is the pullout resistance provided by the self-supporting action of soil. The expression is:

4 $$ \left\{ \begin{gathered} T_{j1} = \eta \pi D(l_{j} - l_{fj} )\cos \alpha \left[ {\gamma h_{aj} + \frac{\sin \alpha }{2}\gamma (l_{j} + l_{fj} ) + q_{0} } \right] \hfill \\ T_{j2} = \pi D(l_{j} - l_{fj} )\sin \alpha \left[ {\gamma h_{aj} + \frac{\sin \alpha }{2}\gamma (l_{j} + l_{fj} ) + q_{0} } \right] \hfill \\ \end{gathered} \right. $$

In the formula: $\eta$ is the friction coefficient, the values can be found in the literature^20^. $D$ is the diameter of the anchorage section. $l_{fj}$ and $l_{j}$ are the free section length and the total length of the *j-th* row of anchors, respectively. $\gamma$ is the unit weight. $q_{0}$ is the additional load on the ground. $h_{aj}$ is the thickness of the overlain soil layer at the calculated point of anchoring section.

### Analysis of earth pressure on retaining structure

**Figure 2:**
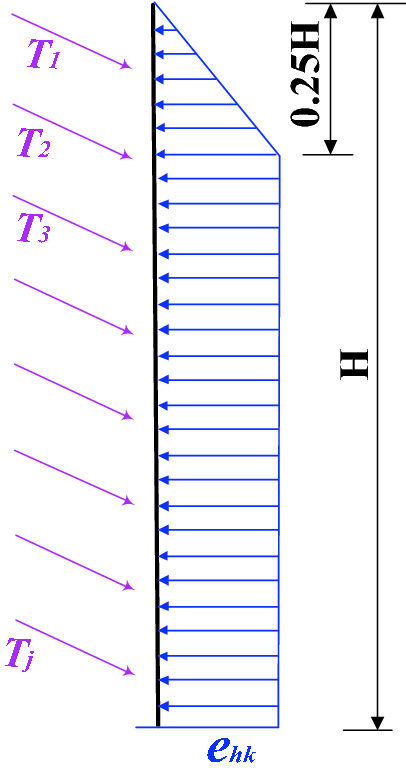
Sketch map of earth pressure.

Based on the earth pressure calculation model of the retaining wall with anchors recommended by “Technical code for building slope engineering” (GB50330-2013)^21^, the lateral pressure distribution of the multi-layer anchor flexible retaining structure is established by considering the factors such as the number of anchor layers, the displacement of retaining wall and the stiffness of retaining structure. As shown in in Fig. 2, for soil slope:

5 $$ e_{hk} = E_{hk} /0.875H $$

where $e_{hk}$ and $E_{hk}$ are the standard value of horizontal component and horizontal resultant force of lateral earth pressure, respectively.

Using Coulomb earth pressure theorem to calculate $E_{hk}$, the internal friction angle $\varphi$ and cohesion $c$ are expressed by equivalent internal friction angle $\varphi_{D}$. The Coulomb earth pressure coefficient $K_{{\text{a}}}$ is:

6 $$ K_{{\text{a}}} = \frac{{\cos^{2} \left( {\varphi_{D} - \beta + \frac{\pi }{2}} \right)}}{{\cos^{2} \left( {\beta - \frac{\pi }{2}} \right)\cos \left( {\beta - \frac{\pi }{2} + \delta } \right)\left[ {1 + \sqrt {\frac{{\sin (\varphi_{D} + \delta )\sin \varphi_{D} }}{{\cos \left( {\beta - \frac{\pi }{2} + \delta } \right)\cos \left( {\beta - \frac{\pi }{2}} \right)}}} } \right]^{2} }} $$

7 $$ \varphi_{D} = \arctan \left( {\tan \varphi + \frac{c}{{\gamma H + q_{0} }}} \right) $$

where $\beta$ is the wall back inclination, $\theta$ is the inclination of the slip surface. $\delta$ is the external friction angle between soil and retaining wall.

The total active earth pressure $P_{a}$ is:

8 $$ P_{a} = \frac{1}{2}\gamma (H + q_{0} /\gamma )^{2} K_{a} $$

The horizontal component of active earth pressure can be expressed as:

9 $$ E_{hk} = P_{a} \cos \left( {\beta - \frac{\pi }{2} + \delta } \right) $$

## Energy analysis of the loess fill slope supported by frame prestressed anchors

The principle of minimum potential energy is a special case of the principle of constant potential energy in the range of linear elasticity. For general problems, the "equilibrium state" of real displacement makes the potential energy of the structural system take the stationary value, that is, the first-order variation becomes zero, and the stable equilibrium state makes the potential energy of the structure take the minimum value, that is, when the potential energy of a system is the minimum, the system will be in a balanced and stable state^16^. As mentioned earlier, the total potential energy $\prod$ of loess fill slope system supported by frame prestressed anchors can be divided into the bending strain energy of frame columns $U_{1}$, the tensile strain energy of prestressed anchors $U_{2}$, the shear strain energy of prestressed anchors $U_{3}$, the tensile external potential energy of anchors $U_{4}$, and the external potential energy of active earth pressure $U_{5}$. They are introduced as follows:

1. Bending strain energy of frame columns.
  10 $$ U_{1} = \frac{1}{2}E_{kz} I_{kz} \int_{0}^{H} {(y^{\prime\prime})^{2} dz = } \frac{{E_{kz} I_{kz} \pi^{4} }}{{16H^{3} }}s^{2} $$
  where $E_{kz} I_{kz}$ is the bending stiffness of the column. $I_{kz}$ is the inertia moment: $I_{kz} { = }ab^{3} /12$, $a$ and $b$ are the section width and height of the frame column, respectively.
2. Tensile strain energy of prestressed anchors.
  11 $$ \begin{aligned} U_{2} & = \sum\limits_{j = 1}^{n} {\left( {\frac{1}{2}k_{Mj} \left( {\frac{y}{\cos \alpha } + \Delta_{j} } \right)^{2} - \frac{1}{2}k_{Mj} \Delta_{j}^{2} } \right)} \\ &  { = }\sum\limits_{j = 1}^{n} {\frac{1}{2}k_{Mj} \left( {\frac{{s^{2} }}{4}\left( {\frac{{\cos \frac{\pi z}{H} - 1}}{\cos \alpha }} \right)^{2} - s\left( {\frac{{\cos \frac{\pi z}{H} - 1}}{\cos \alpha }} \right)\Delta_{j} } \right)} \\ \end{aligned} $$
  where $n$ is the number of rows of the anchor. $k_{Mj}$ is the stiffness of the anchor: $k_{Mj} = E_{a} A/l_{fj}$, $A$ and $E_{a}$ are cross section area and elastic modulus of the anchor, respectively. $\Delta_{j}$ is the initial deformation of the *j-th* row of prestressed anchors: $\Delta_{j} = p/k_{Mj}$, $p$ is the applied prestress.
3. Shear strain energy of prestressed anchors.
  Under the action of self-weight and additional load on the upper part, the filling body of loess fill slope will produce relative displacement at the potential slip surface, and the prestressed anchor is subjected to shear action (not considering the torsion action of anchor rod). According to the mechanical equilibrium^19^, the support force perpendicular to the axial direction of the prestressed anchor can be expressed as:
  12 $$ F_{Nj} = (\gamma h_{fj} + q_{0} )\cos \alpha $$
  where $h_{fj}$ indicates the thickness of the overlying soil layer of the anchor at the slip surface.
  13 $$ U_{3} = \sum\limits_{j = 1}^{n} {\int_{{l_{s} }} {\frac{{(F_{Nj} )^{2} }}{2GA}} dx = } \sum\limits_{k = 1}^{m} {\sum\limits_{j = 1}^{n} {\frac{{(F_{Nj} )^{2} }}{2GA}l_{s} } } $$
  where $G$ is the shear modulus of the anchor rod: $G = E_{a} /[2(1 + \mu_{a} )]$, $\mu_{a}$ and $E_{a}$ are the Poisson's ratio and elastic modulus of the anchor, respectively. $l_{s}$ is the free section length of the anchor after deformation:
  14 $$ l_{s} = l_{fj} + \frac{y}{\cos \alpha } + \Delta_{j} $$
  Thus:
  15 $$ U_{3} = \sum\limits_{j = 1}^{n} {\frac{{(F_{Nj} )^{2} }}{2GA}(l_{fj} + \Delta_{j} )} - \sum\limits_{j = 1}^{n} {\frac{{(F_{Nj} )^{2} }}{2GA}\frac{{\cos \frac{\pi z}{H} - 1}}{2\cos \alpha }s} $$
4. Tensile external potential energy of anchors.
  16 $$ U_{4} = - \sum\limits_{j = 1}^{n} {T_{j} \cdot (y/\cos \alpha )} { = }\frac{s}{2}\sum\limits_{j = 1}^{n} {T_{j} \cdot \frac{{\cos \frac{\pi z}{H} - 1}}{\cos \alpha }} $$
5. External potential energy of active earth pressure.
  17 $$ \begin{aligned} U_{5} & = - \int_{0}^{H} {e_{hk} ydz} = - \left( {\int_{0.75H}^{H} {\frac{{e_{hk} (H - z)y}}{0.25H}dz} + \int_{0}^{0.75H} {e_{hk} ydz} } \right) \\ &  = - \left( {\frac{7}{16} + \frac{\sqrt 2 - 2}{{\pi^{2} }}} \right)e_{hk} Hs \\ \end{aligned} $$

Based on the above analysis, the total potential energy $\prod$ of the fill loess slope system supported by frame prestressed anchors is:

18 $$ \prod = U_{1} + U_{2} + U_{3} + U_{4} + U_{5} $$

According to the minimum potential energy method^16^: $\frac{\partial \prod }{{\partial s}} = 0$, we can get:

19 $$ s = \frac{{\frac{1}{2}\sum\nolimits_{j = 1}^{n} {k_{{M_{j} }} \left( {\frac{{\cos \frac{\pi z}{H} - 1}}{\cos \alpha }} \right)\Delta_{j} + \left( {\frac{7}{16} + \frac{\sqrt 2 - 2}{{\pi^{2} }}} \right)He_{hk} } }}{{\frac{{E_{k} I_{k} \pi^{4} }}{{8H^{3} }} + \frac{1}{4}\sum\nolimits_{j = 1}^{n} {k_{M} \left( {\frac{{\cos \frac{\pi z}{H} - 1}}{\cos \alpha }} \right)^{2} } }} + \frac{{ - \frac{1}{2}\sum\nolimits_{j = 1}^{n} {T_{j} \frac{{\cos \frac{\pi z}{H} - 1}}{\cos \alpha } + \sum\nolimits_{j = 1}^{n} {\frac{{(F_{Nj} )^{2} }}{2GA}\frac{{\cos \frac{\pi z}{H} - 1}}{2\cos \alpha }} } }}{{\frac{{E_{k} I_{k} \pi^{4} }}{{8H^{3} }} + \frac{1}{4}\sum\nolimits_{j = 1}^{n} {k_{M} \left( {\frac{{\cos \frac{\pi z}{H} - 1}}{\cos \alpha }} \right)^{2} } }} $$

The horizontal displacement of loess fill slope supported by frame prestressed anchors at any calculated height can be obtained by formula (2) and formula (19).

## Example analysis and verification

### Engineering example

**Figure 3:**
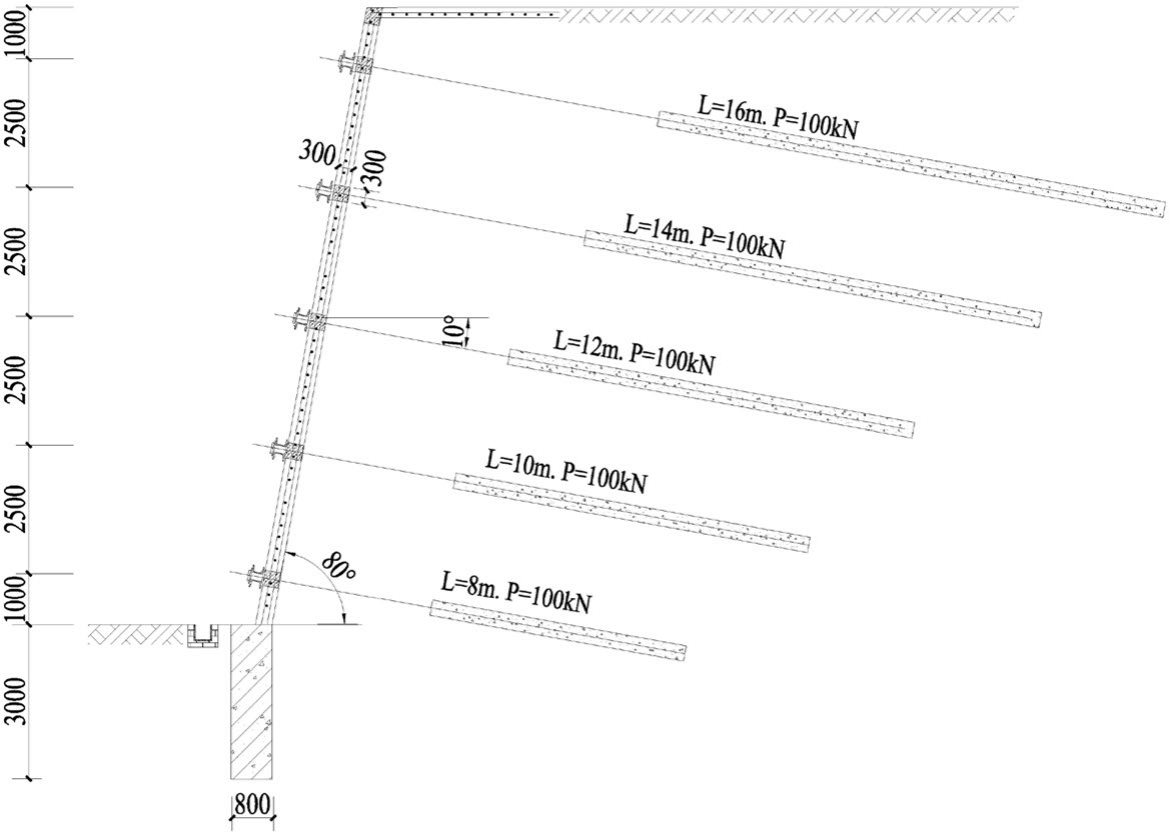
Design result of slope support.

A fill slope with a height of 12 m and a gradient of 80 degrees is supported by frame prestressed anchors. The soil parameters are shown in Table 1. The supporting scheme is shown in Fig. 3. The specific design parameters are shown in Table 2, the section size of column is 300 mm × 300 mm. The load on the top of the slope is 20 kPa, the number of anchor rows $n = 5$, and the angle of the anchor is 10 degrees. The calculation parameters are given in Table 3^22,23^.

**Table 1:** Parameters of soil.

Soil	$\gamma$(kN/m^3^)	$c$(kPa)	$\varphi$(°)	$\mu$	$E_{s}$(MPa)	$\tau$(kN/m^2^)
Loess	19	35	27	0.30	28	50

$\gamma$ unit weight, $\varphi$ internal friction angle, $c$ cohesion, $E_{s}$ elastic modulus, $\mu$ Poisson’s ratio, $\tau$ ultimate frictional.

**Table 2:** Design results of anchors.

$z$	$s_{H}$	$s_{V}$	$D$	$d$	$l_{f}$	$l_{a}$	$l$
11	2.5	2.5	150	28	6	10	16
8.5	2.5	2.5	150	28	5	9	14
6	2.5	2.5	150	28	4	8	12
3.5	2.5	2.5	150	28	3	7	10
1	2.5	2.5	150	28	3	5	8

$z$ anchor height, $s_{V}$ vertical spacing of anchors (m), $s_{H}$ horizontal spacing of anchors (m), $d$ diameter of reinforcement, $D$ diameter of anchorage (mm)., $l_{f}$ length of the free section of the anchor (m), $l_{a}$ length of anchoring section of the anchor (m), $l$ total length of the anchor (m).

**Table 3:** Calculation parameters.

$\eta$	$E_{a}$($N/mm^{2}$)	$G$($N/mm^{2}$)	$E_{k}$($N/mm^{2}$)
0.38	$2 \times 10^{5}$	$0.77 \times 10^{5}$	$3.5 \times 10^{4}$

$\eta$ friction coefficient of the anchor, $E_{a}$ elastic modulus of the anchor, $G$ shear modulus of the anchor, $E_{k}$ elastic modulus of the frame column.

### Method validation

**Figure 4:**
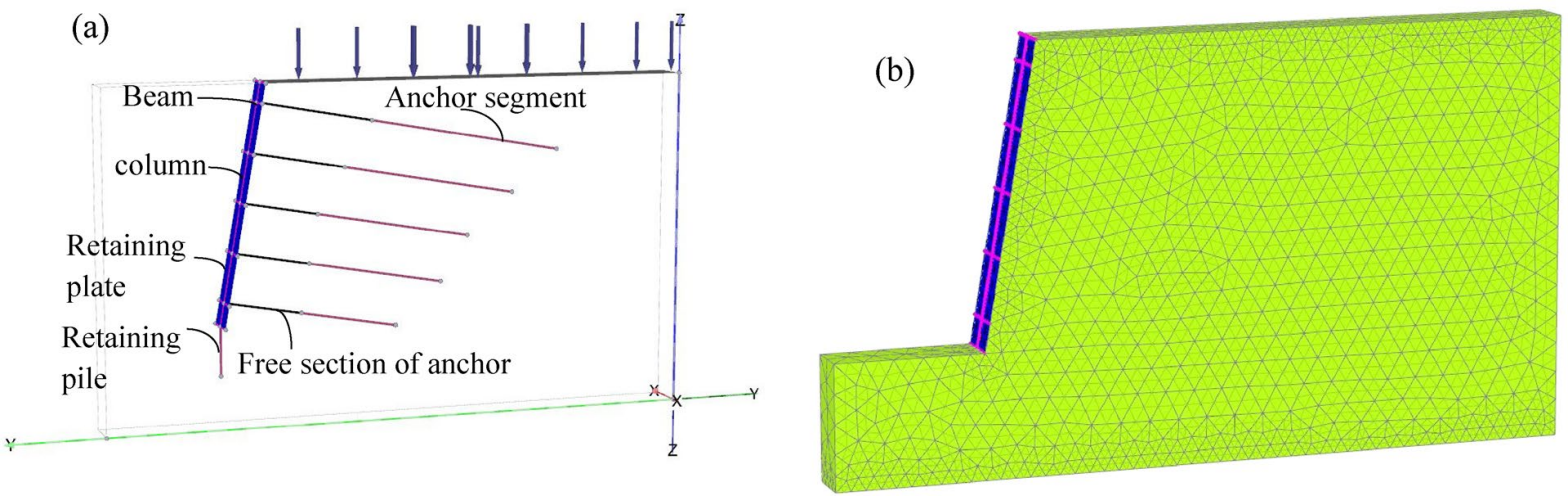
Finite element numerical model: (**a**) Model establishment (**b**) Mesh division.

To verify the rationality of the method proposed in this paper, a numerical model of “Engineering example” was established by PLAXIS 3D finite element software (Fig. 4). The supporting structure in the model adopts linear elastic model, the frame (beam + column) is simulated by beam element, the retaining plate is simulated by plate element, and the free section of anchor is simulated by point-to-point anchor element, the embedded pile element is used to simulate the retaining pile and anchor segment^24,25^. The parameters of the supporting structure are shown in Table 4. The surrounding boundary of the model is a normal fixed boundary, complete constraint at the bottom and free boundary at the top^26^. The soil is modeled by SSC consolidation creep model^2^, and the soil parameters are obtained according to “Engineering example” and relevant empirical formulas^27^, as shown in Table 5. The initial stress field of the model is considered according to the self-weight stress of the foundation, the calculation type is "K_0_ process", and the loading type is "staged construction". After that, the calculation type is "consolidation".

**Table 4:** Parameters of the supporting structure.

Parameters	Beams, columns	Free section of the anchor	Anchoring section of the anchor	Retaining plates
E (kN/m^2^)	3.5 × 10^7^	2 × 10^8^	2.0 × 10^5^	8 × 10^6^
γ (kN/m^3^)	25	–	28	20

**Table 5:** Parameters of soil.

$\gamma$/(kN/m^3^)	$\gamma_{sat}$(kN/m^3^)	c/(kPa)	$\varphi$/(°)	E/(MPa)	$\mu$	$\lambda^{*}$	$\mu^{*}$	$\kappa^{*}$	$k_{z}$/(m/d)
19.0	19.84	35	27	30	0.26	0.0689	0.0023	0.0069	0.0024

$\gamma$ unit weight, $\gamma_{sat}$ saturated unit weight, $c$ cohesion, $\varphi$ internal friction angle, $\mu$ poisson ratio, $E$ elastic modulus, $\lambda^{*}$ modified compression index, $\mu^{*}$ modified creep exponent, $\kappa^{*}$ modified rebound coefficient, $k_{z}$ permeability coefficient, in this paper, $k_{z} /k_{x} = 10$ ($k_{y} { = }k_{x}$) taken to calculate^27^.

**Figure 5:**
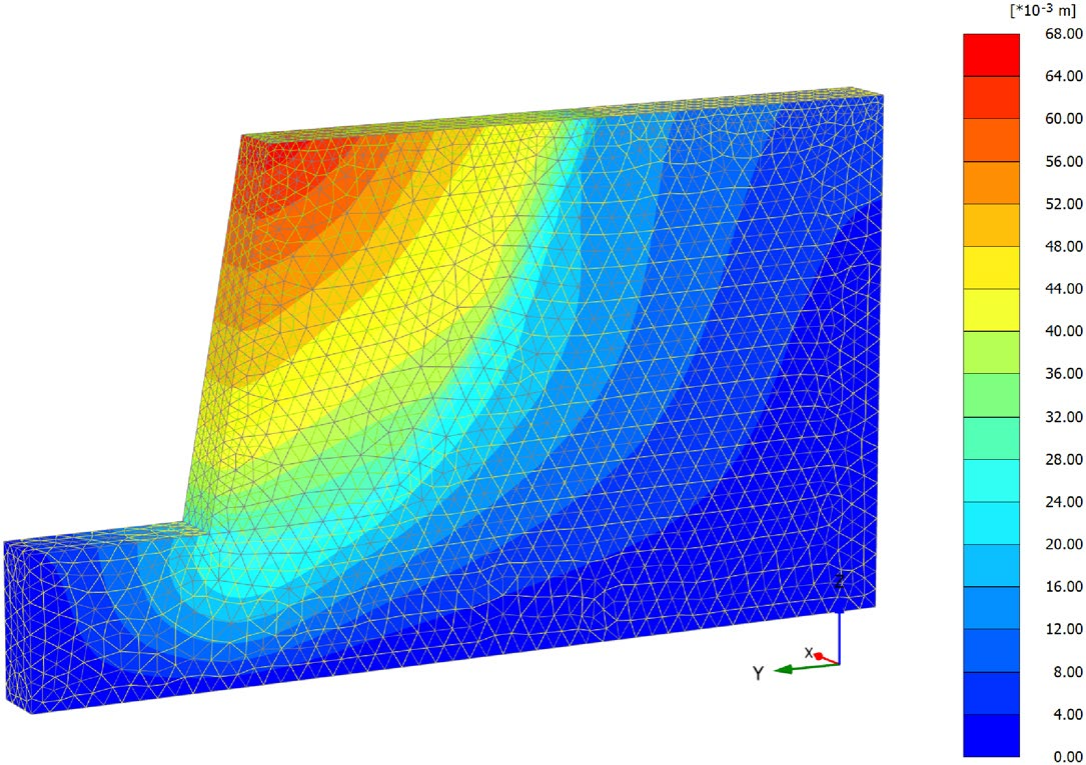
Deformation nephogram of unsupported slope.

**Figure 6:**
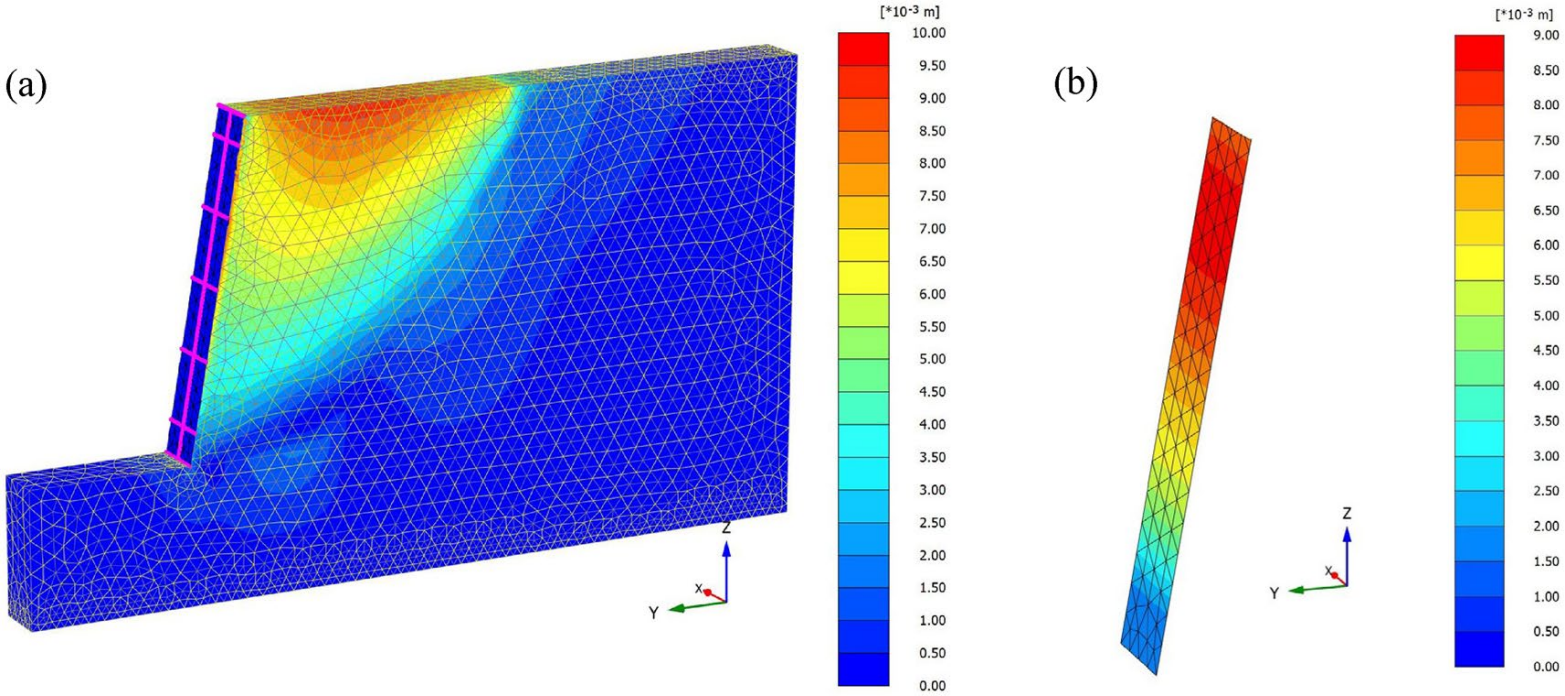
Deformation nephogram of supported slope: (**a**) Slope deformation (**b**) Slope surface deformation.

Figures 5 and 6 show the displacement nephogram of loess fill slope without support and with support, respectively. We can see that the vertical settlement of the soil under the action of self-weight drives the soil to move to the free surface of the slope. After support, the displacement is significantly constrained.

According to the deformation nephogram, the unsupported slope shows integral sliding deformation, and the maximum displacement occurs at the slope shoulder, with a value of 68 mm (Fig. 6a). However, the slope displacement after supporting has been greatly improved. Under the constraint of supporting structure, the maximum displacement at the top of the slope occurs at a certain distance from the slope shoulder. It can be seen from Fig. 6b that the distribution law of the horizontal displacement of the slope surface is larger at the upper part and smaller at the lower part, which is the same as the distribution trend of the horizontal displacement calculation method proposed in this paper. The displacement from the top to the bottom of the slope decreases stepwise, and the maximum horizontal displacement is 9.18 mm, which is located at the slope shoulder.

The calculated results of the method presented in this paper are compared with those of numerical simulation, as shown in Table 6. Compared with the calculation results of the method proposed in this paper, it is found that the numerical simulation results are smaller, this may be related to the difference between actual and selected earth pressures for calculation, resulting in a certain difference in results, but the displacement distribution trend is consistent on the whole, which shows that the calculation method in this paper is reliable.

**Table 6:** Calculation results.

Calculated position $z$(m)	1	3.5	6	8.5	11
**Horizontal displacement **$y$** (mm)**					
Methods of this paper	0.18	2.15	5.51	8.85	10.81
Numerical simulation	0.12	3.25	6.53	7.89	9.18

**Figure 7:**
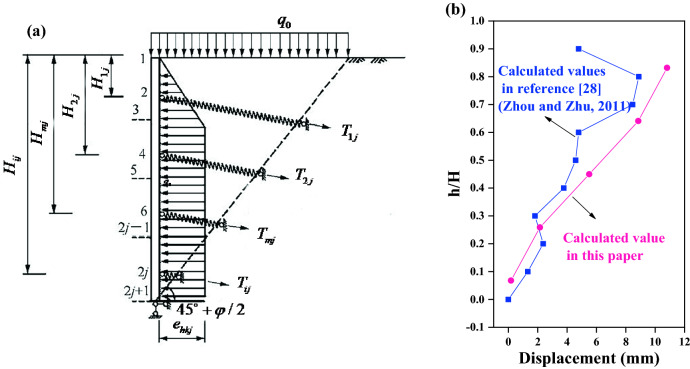
Comparison of theoretical calculation of horizontal displacement^28^ (**a**) Computational model; (**b**) Displacement curve.

Considering the influence of the construction process of the foundation pit slope, a method for calculating the horizontal displacement of the slope surface of the frame prestressed anchor flexible supporting structure is presented in reference^28^, the calculation model of earth pressure is chosen as trapezoid, and the free segment of anchor is assumed to be spring. The flexible retaining structure of frame with prestressed anchor rod is simplified as a continuous beam with bottom hinged and upper supported by anchor at different supporting heights, the total displacement of the slope considering the superposition of distributed excavation is calculated according to the schematic diagram shown in Fig. 7a.

The method proposed in this paper is compared with the method presented in reference^28^, which is shown in Fig. 7b. Where H/h is the ratio of the height of the calculated point to the height of the slope, the horizontal displacement distribution obtained by this method is similar to that calculated in reference^28^: the upper displacement is larger, the lower displacement is smaller. In numerical value, the horizontal displacement calculated by the method of minimum potential energy is slightly larger than that calculated by considering the construction process. On the whole, the calculated results in this paper are in good agreement with those in reference^28^.

**Figure 8:**
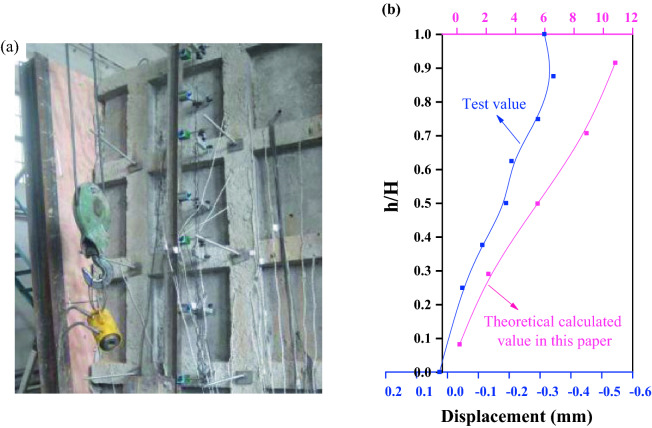
Horizontal displacement of slope supported by frame prestressed anchors^18^ (**a**) Indoor test; (**b**) Displacement curve.

The experimental results in reference^18^ are used to verify the algorithm in this section. In order to deeply understand the working performance of the flexible supporting structure of frame prestressed anchors, especially the displacement control function of the flexible supporting structure, Zhou and Zhu (2010) designed and completed the model test of the loess slope supported by frame prestressed anchors (Fig. 8a), and analyzed the distribution law of the horizontal displacement of the slope.

According to the test results, the distribution curve of the horizontal displacement along the height direction is obtained as shown in Fig. 8b, where h/H is the ratio of the height of the measuring point to the height of the slope, and the negative displacement is the displacement which points to the free surface. Figure 8b also shows the distribution curve of the calculated results in this paper. It is necessary to explain that, as the test is a basic study, the model test is carried out for geotechnical problems and is restricted by the test conditions, the test model is not designed strictly according to the similarity method, and the geometric similarity of the model is mainly considered. Therefore, the test results can only be qualitatively analyzed, and cannot quantitatively express the actual deformation of the original slope. The test results show that the displacement of the slope is non-linear, and the displacement of the middle and upper part of the supporting structure is larger and increases gradually along the slope height. The displacement distribution is consistent with the results in this paper, which confirms the validity of the flexible deformation model assumption in this paper.

To sum up, the calculation method of horizontal displacement of loess fill slope supported by frame prestressed anchors based on energy method proposed in this paper is reasonable.

### Parametric analysis

This section mainly analyzes the influence of soil parameters and supporting structure parameters on the horizontal displacement of loess fill slope supported by frame prestressed anchor. The parameters of soil include unit weight, internal friction angle and cohesion. The parameters of supporting structure include prestress, diameter of anchor and inclination of anchor.

(1) Influence of soil parameters on horizontal displacement.

**Figure 9:**
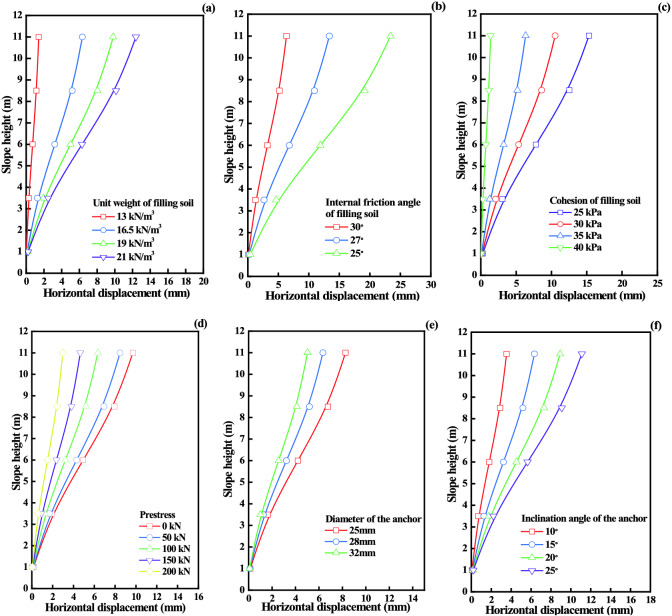
Influence of parameter change on horizontal displacement: (**a**) Unit weight. (**b**) Internal friction angle. (**c**) Cohesion. (**d**) Prestress of the anchor. (**e**) Diameter of the anchor. (**f**) The inclination angle of the anchor.

The internal friction angle, cohesion and unit weight of soil are important parameters for the design of frame-supported structures with prestressed anchors. As shown in Fig. 9a–c, the displacement of the slope gradually increases with the increase of the unit weight of soil, but the increasing rate decreases gradually. With the decrease of internal friction angle, the displacement and growth rate of slope increase. With the increase of cohesion, the slope displacement decreases gradually. In the calculation, the internal friction angle, cohesion and unit weight of soil are related to the equivalent internal friction angle, which affects the active earth pressure of the soil behind the slope, so it has a great influence on the horizontal displacement. This also shows that for fill slopes, the selection of fillers has a significant impact on slope displacement. In the actual filling project, the filling material is usually taken locally, and the compactness and moisture content of the filling soil should be controlled to minimize the later consolidation settlement deformation of the filling body.

(2) Influence of supporting structure parameters on horizontal displacement.

The design parameters of supporting structure have a significant impact on slope displacement. Changing the prestress of the anchor, it can be seen from Fig. 9d that the maximum displacement of slope top is 9.7 mm without prestress (*p* = 0 kN). When prestress is applied to 100 kN, the maximum displacement of slope top is 6.34 mm, and the displacement is reduced by 35%. This shows that the application of prestress can effectively control the slope displacement. The supporting structure of frame prestressed anchors belongs to active flexible supporting structure. Without prestress, the supporting structure does not enter the active supporting state, which is the traditional passive support. After loading prestress, the supporting state changes. With the increase of prestress, the slope displacement decreases gradually. In engineering construction, the prestress applied value should be reasonably selected within the allowable range of design, so as to realize the advanced control of slope deformation.

Changing the diameter of the anchor, as shown in Fig. 9e, the larger the anchor diameter, the smaller the slope displacement. According to the calculation formula, the initial deformation of the anchor decreases with the increase of the diameter of the anchor. The tensile stiffness of the anchor increases and the pullout force increases, which provides an effective constraint for the slope displacement.

Changing the angle of the anchor, as shown in Fig. 9f, the displacement increases with the increase of the angle of the angle. According to the mechanism of the anchor, the greater the angle of the anchor is, the smaller the tension force in the horizontal direction of the anchor is, and the smaller the constraint on the horizontal displacement is, resulting in the gradual increase of the displacement.

## Conclusions

The calculation method of horizontal displacement of slope considering the deformation characteristics of flexible retaining structure is put forward, and the following conclusions are obtained through calculation and analysis.

1. The displacement calculation method of loess fill slope supported by frame prestressed anchors is put forward, and the analytical solution of horizontal displacement of slope is derived, and its rationality is verified by practical engineering application and numerical simulation.
2. The minimum potential energy method combined with the characteristics of flexible retaining structure to solve the horizontal displacement of loess fill slope supported by frame prestressed anchor is clear in concept and simple in solving method, which can be applied to the optimization of displacement control scheme of flexible retaining fill slope.
3. According to the results of parameter analysis, the filling parameters have great influence on the horizontal displacement, so the quality of filling should be controlled in practice. In addition, the pre-stress of anchor can control the horizontal displacement of slope remarkably. The larger the prestress is, the smaller the slope displacement is. Therefore, the prestress should be reasonably selected in the design of supporting structure.
4. The calculation method proposed in this paper can be applied to the structural optimization design of loess fill slope supported by frame prestressed anchors, and further enrich the displacement calculation theory of slope supported by flexible retaining structure.

## References

[1] Xie X, Qi S, Zhao F, Wang D (2018). Creep behavior and the microstructural evolution of loess-like soil from xi'an area, China. Eng. Geol..

[2] Zhu C, Li N (2019). Ranking of influence factors and control technologies for the post-construction settlement of loess high-filling embankments—ScienceDirect. Comput. Geotech..

[3] Zhao Z, Zhu Y, Ye S (2021). Study on settlement deformation of high fill foundation in large thickness loess area. Arab. J. Geosci..

[4] Carey JM, Cosgrove B, Norton K, Massey CI, Lyndsell B (2021). Debris flow-slide initiation mechanisms in fill slopes, wellington, New Zealand. Landslides.

[5] Wang C, Wang B, Guo P, Zhou S (2015). Experimental analysis on settlement controlling of geogrid-reinforced pile-raft-supported embankments in high-speed railway. Acta Geotech..

[6] Wang J, Xu Y, Ma Y, Qiao S, Feng K (2018). Study on the deformation and failure modes of filling slope in loess filling engineering: A case study at a loess mountain airport. Landslides.

[7] Huang A, Ye S (2020). Sensitivity of high fill slope stability factors under seismic conditions. Soil Mech. Found. Eng..

[8] Ye S, Huang A (2020). Sensitivity analysis of factors affecting stability of cut and fill multistage slope based on improved grey incidence model. Soil Mech. Found. Eng..

[9] Muething N, Zhao C, Hoelter R, Schanz T (2018). Settlement prediction for an embankment on soft clay. Comput. Geotech..

[10] Wang L, Shao S, She F (2020). A new method for evaluating loess collapsibility and its application. Eng. Geol..

[11] Goh ATC, Zhang R, Wang W, Wang L, Liu H, Zhang W (2020). Numerical study of the effects of groundwater drawdown on ground settlement for excavation in residual soils. Acta Geotech..

[12] Huang Q, Xu X, Kulatilake P, Lin F (2020). Formation mechanism of a rainfall triggered complex landslide in southwest china. J. Mt. Sci..

[13] Ye S, Zhao Z (2020). Allowable displacement of slope supported by frame structure with anchors under earthquake. Int. J. Geomech..

[14] Ye S, Zhao Z (2020). Seismic response of pre-stressed anchors with frame structure. Math. Problems Eng..

[15] Xu X, Chen S, Xu H (2006). Spatial deformation analysis of cantilever soldier pile retaining structure in deep foundation pit. Rock Soil Mech..

[16] Stephen P. Timoshenko. Theory of Elastic Stability[M]. New YMcGRAW-HILL BOOK COMPANY, Inc (1961).

[17] Dong J, Zhu Y, Zhou Y, Ma W (2010). Dynamic calculation model and seismic response for frame supporting structure with prestressed anchors. Sci. China Technol. Sci..

[18] Zhou Y, Zhu Y (2010). Theoretical analysis and model test study of slope horizontal displacement of grillage flexible supporting structure with prestressed anchors. Chin. J. Rock Mech. Eng..

[19] Zhou Y, Zhu Y (2012). Research on anti-pulling force of anchor of flexible supporting system with prestressed anchors. Rock Soil Mech..

[20] Chen Z, Cui J (2000). Application of Soil Nailing in Foundation Pit Engineering.

[21] Technical code for building slope engineering (GB50330-2013)

[22] Ye S, Zhao Z, Zhu Y (2019). Large-scale shaking table model test of loess slope supported by frame anchors. Rock Soil Mech..

[23] Ye S, Fang G, Ma X (2019). Reliability analysis of grillage flexible slope supporting structure with anchors considering fuzzy transitional interval and fuzzy randomness of soil parameters. Arab. J. Sci. Eng..

[24] Zhang W, Wu C, Li Y, Wang L, Samui P (2019). Assessment of pile drivability using random forest regression and multivariate adaptive regression splines. Georisk..

[25] Zhang W, Li Y, Goh ATC, Zhang R (2020). Numerical study of the performance of jet grout piles for braced excavations in soft clay. Comput. Geotech..

[26] Zhang R, Zhang W, Goh ATC (2018). Numerical investigation of pile responses caused by adjacent braced excavation in soft clays. Int. J. Geotech. Eng..

[27] Zhou Y, Wu H, Zhu C, Li N (2018). Sensitivity analysis of influence factors of post-construction settlement on loess high fill embankment. J. Xi’an Univ. Technol..

[28] Zhou Y, Zhu Y (2011). Influencing factors of horizontal displacement of wall facing of grillage flexible supporting structure with prestressed anchors. Chin. J. Geotech. Eng..

